# Sparsity-Based Spatial Interpolation in Wireless Sensor Networks

**DOI:** 10.3390/s110302385

**Published:** 2011-02-25

**Authors:** Di Guo, Xiaobo Qu, Lianfen Huang, Yan Yao

**Affiliations:** Department of Communication Engineering, Xiamen University, Xiamen 361005, China; E-Mails: guodi@xmu.edu.cn (D.G.); quxiaobo@xmu.edu.cn (X.Q.); yaoy@tsinghua.edu.cn (Y.Y.)

**Keywords:** data interpolation, sparsity, wireless sensor network

## Abstract

In wireless sensor networks, due to environmental limitations or bad wireless channel conditions, not all sensor samples can be successfully gathered at the sink. In this paper, we try to recover these missing samples without retransmission. The missing samples estimation problem is mathematically formulated as a 2-D spatial interpolation. Assuming the 2-D sensor data can be sparsely represented by a dictionary, a sparsity-based recovery approach by solving for *l*_1_ norm minimization is proposed. It is shown that these missing samples can be reasonably recovered based on the null space property of the dictionary. This property also points out the way to choose an appropriate sparsifying dictionary to further reduce the recovery errors. The simulation results on synthetic and real data demonstrate that the proposed approach can recover the missing data reasonably well and that it outperforms the weighted average interpolation methods when the data change relatively fast or blocks of samples are lost. Besides, there exists a range of missing rates where the proposed approach is robust to missing block sizes.

## 1. Introduction

A wireless sensor network (WSN) typically consists of a potentially large number of wireless devices able to take environmental measurements [1]. Typical examples of such environmental measurements include temperature, light, sound, and humidity [2,3]. These sensor readings are then directly transmitted over a wireless channel to a central node [4], called the sink, where a running application makes decisions based on these sensor readings.

**Figure 1.**
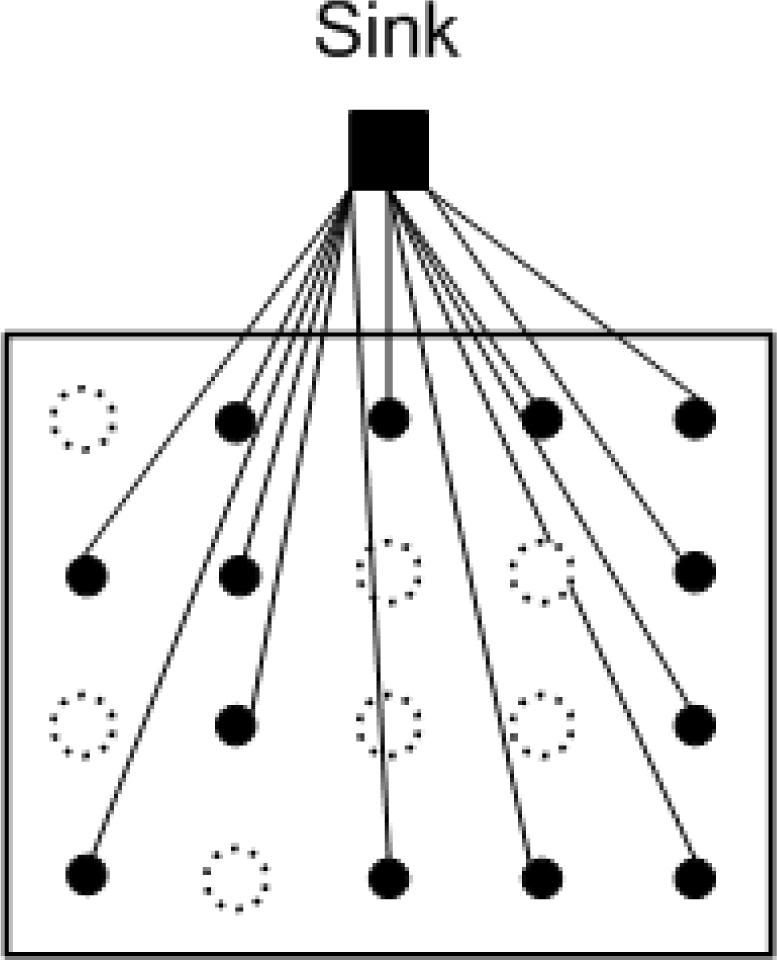
A sensor network with missing samples. “ 
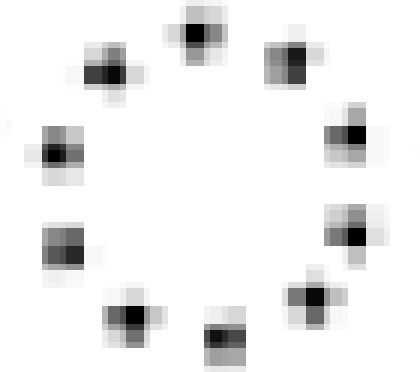
” represents an unsampled location.

The fusion of information from multiple sensors with different physical characteristics enhances the understanding of our surroundings and provides the basis for planning, decision-making, and control of autonomous and intelligent machines [5]. Unfortunately, due to factors such as packet loss and collisions, low sensor battery levels, and potential harsh environmental conditions [6], not all sensor readings can be successfully gathered at the sink, *i.e.*, some readings could be lost. Often, the sensors are simple devices that do not support retransmission and furthermore, the strict energy constraints of sensor nodes also result in great limitations for number of transmissions. In other cases, the retransmission may not be possible when the sensors are permanently broken. Figure 1 shows a large scale WSN with missing samples. Large scale WSNs are known to suffer from coverage holes, *i.e.*, regions of the deployment area where no sensing coverage can be provided [7]. Such holes are often the result of network congestion, hardware failures, extensive costs for deployment, or the hostility of deployment areas. Lee and Jung [8] have proposed an adaptive routing protocol to recover a network after failures after large areas, Peng [9] improved the accuracy of node fault detection when number of neighbor nodes is small and the node’s failure ratio is high.

In this paper, we aim to reasonably recover the missing data without retransmission. Due to the nature of the network topology, readings among sensors may be strongly correlated. This correlation provides us a good opportunity to recover these missing samples. For example, Collins *et al*. [10] and Sheikhhasan [11] have discussed temperature interpolation with the help of spatial correlations.

Roughly speaking, there are two typical ways to investigate the spatial correlation for data interpolation or missing data recovery, which are inverse distance weighted averaging (IDWA) [10,11] and Kriging [7,10].

The inverse-distance weighted averaging (IDWA), which is relatively fast and easy to compute, is one of the most frequently used methods in the spatial interpolation [12–14]. Assuming the spatial correlation in adjacent sensors is uniform, IDWA tries to estimate the values of unsampled sensors in the form of some linear combination of values at neighboring known sampled sensors. The weights for the linear combination only depend on the distance between the unsampled and the sampled sensors [12–14]. The sensors located close to the unsampled sensors are assigned larger weights than the sensors that are far away from the unsampled sensors. Thus, IDWA will work well if the values of unsampled sensors are expected to be similar to values of the neighboring sensors. However, this assumption affects the estimation accuracy in many practical situations, where physical phenomena evolve in a more erratic way than uniformly increasing or decreasing in magnitude [7]. The averaging process in IDWA has the tendency to smoothen the data, which is not suitable for the situation when data change fast in the area of interest. In addition, for the special case that a block of sensors are missing, IDWA may not provide a confident estimation since the measurements beyond the missing-block may be very different from the measurements within the missing-block.

Kriging [7,10] is another way to estimate the missing samples using the combination of available measurements. By calculating the spatial correlation between two points, a semivariogram is defined to obtain the weights for linear combination. As a result, these weights vary spatially and depend on the correlation. Assuming the historical variogram is known and can approximately represent the current variogram, missing samples are estimated based on the historical variogram function. However, the spatial interpolation may not be right if the semivariogram varies a lot in the temporal dimension.

In this paper, we propose a sparsity-based recovering method that can capture the spatial variation and does not require knowledge of the historical spatial correlation. Suppose a wireless sensor network is deployed to monitor a certain spatially varying phenomenon such as temperature, light, or moisture, a snapshot of the physical field being measured can be viewed as a signal or image with some degree of spatial correlation [7]. If the sensors are geographically placed in a uniform fashion, then 2-D Discrete Cosine Transform (DCT) or 2-D Discrete Wavelet Transform (DWT) can be used to sparsify the network data. The fast changes in a local region often can be sparsely represented as some high frequency components and the smooth region can be represented by some low frequency components. As an exciting research topic in signal processing, compressed sensing (CS) was introduced by Bajwa *et al.* into wireless networks [15]. Haupt *et al.* gave a comprehensive review and looked forward to the prospect of CS in sensor networks [16]. Lu *et al*. [17] proposed a distributed sparse sampling algorithm to efficiently estimate the unknown sparse sources in a diffusion field.

The main difference between the missing data recovery problem and the conventional compressive sensing (CS) is that in the conventional CS, the sampling scheme can be designed by the users, and usually random linear projections are preferred, while in the missing data recovery problem the sampling matrix cannot be controlled by the user since it is determined by the missing events, e.g., locations of missing nodes in the network.

In this paper, assuming the sensor data is sparse in the DCT or DWT domain, we propose a sparsity-based spatial interpolation method for recovering missing samples in wireless sensor networks without retransmission. The main contributions of the paper are summarized as follows:

1. A sparsity-based recovery algorithm via solving the *l*_1_ norm minimization to recover the missing samples in the spatial domain is proposed.
2. Based on the theoretical analysis of the proposed method, we discuss how to choose an appropriate dictionary to reduce estimation errors. From a practical point of view, if 2-D sensor data are both sparse in both the DCT and wavelets domains, then DCT is a better choice because a localized basis cannot carry enough information or even no information if the a relative large missing block overlaps with the compact support of basis, e.g., wavelets basis. This is verified by simulations on real data.
3. Extensive comparisons of the proposed method and a weighted average interpolation method called K-Nearest Neighbors (KNN) are conducted. The advantage of the proposed method is demonstrated in terms of criteria root mean square error (RMSE) and visual data fidelity, both on synthetic and real data. Simulations show that using the proposed method one can provide more reasonable recovered data when the data changes fast or missing blocks are large.

Currently, we focus on the regular grid sensor networks. For irregular grid networks, traditional sparsifying transforms, e.g., DCT, may not be applied directly. However, one can also extend the sparsity-based interpolation method to irregular grid sensor network by partitioning the sensors into cells with some tree-structure, e.g., k-d trees [18].

The remainder of this paper is organized as follows. In Section 2, the theoretical framework is developed to define the 2-D missing data recovery problem based on the data sparsity, the recovery error is computed, and how to choose appropriate dictionary to reduce recovery error is also discussed. In Section 3, the advantage of the proposed approach over traditional interpolation methods are illustrated in two examples. In Sections 4, the iterative thresholding algorithm is explored for recovering the missing samples. In Section 5, simulations of missing data recovery are presented for both on synthetic and real data sets. Also, the relationship among the recovery error and the missing pattern is discussed. Advantage of DCT over wavelets for sparsity-based interpolation is demonstrated in Subsection 5.3. Finally, the conclusions are given in Section 6.

## 2. Problem Formulation

Consider that the values *Z*(*x*_1_),*Z*(*x*_2_),⋯*Z*(*x_n_*) represent readings of a spatial process *Z* at locations *x*_1_,*x*_2_,⋯*x_n_* at a given time instant, and they can be collected and arranged in a vector **f** = [*Z*(*x*_1_),*Z*(*x*_2_),⋯*Z*(*x_n_*)]*^T^* to form the network data. The network data **f** ∈ ℝ^*n*^ is assumed to be composed as a linear combination of few atoms from a dictionary **Φ** ∈ ℝ^*n*×*d*^, *i.e.,*:

(1) f=Φx

where **x** ∈ ℝ*^d^* is expected to be sparse, ‖**x**‖_0_; ≪ *n*. The dictionary **Φ** is the *n* × *d* matrix with rank(**Φ**) = *n* ≤ *d*. The dictionary is said to be redundant or overcomplete whenever *n* < *d*.

The network data **f** contains the available data **f***_a_* ∈ ℝ*^m^* and the missing data **f***_p_* ∈ ℝ^*n*−*m*^. After reordering:

(2) $$f=\left[\begin{array}{c} f_{a}\\ f_{p} \end{array}\right],\;\;\;\;f_{a}\in {\mathbb{R}}^{m},\;f_{p}\in {\mathbb{R}}^{n-m}$$

According to the indices of the available data **f***_a_* and the missing data **f***_p_*, the rows of **Φ** are partitioned into two parts as:

(3) $$\Phi =\left(\begin{array}{c} A\\ B \end{array}\right),\;\;\;\;A\in {\mathbb{R}}^{m\times d},\;\;\;\;B\in {\mathbb{R}}^{(n-m)\times d}$$

With this partition, the Equation (2) can be regrouped as:

(4) $$\Phi \text{x}=f\Rightarrow \{\begin{array}{c} \text{Ax}=f_{a}\\ \text{Bx}=f_{p} \end{array}$$

To recover **f**, we can find the solution x^*^ first by solving:

(5) $$\text{Ax}=f_{a}$$

and then plug it into:

(6) $$\text{Bx}=f_{p}$$

to get **f_*p*_**. However, Equation (5) is under-determined since *m* < *d*, thus more than one solutions are possible to satisfy it. Since **x** is sparse, we can employ sparsity to regularize the solution by solving the ℓ_1_;-minimization problem:

(7) $$\text{arg}\;\underset{\text{x}}{\text{min}}{\left\|\text{x}\right\|}_{1}\;\;\text{s.t.}\;\text{Ax}=f_{a}$$

Now suppose **f** is *k* -sparse, *i.e.*, it can be represented as a weighted combination of *k* columns of dictionary **Φ**. Given the support of coefficient vector **x** is *S*_x_ = {*i:x_i_* ≠ 0}, the cardinality of **x**:|*S***_x_**| = *k*, and *S***_x_** is the set of index of nonzero entry in **x**, accordingly, the available data is:

(8) $$f_{a}=\sum_{j\in S_{\text{x}}}x_{j}A_{j}$$

where *A_j_* stands for the *j*th column of **A**. For simplicity, we assume all columns of **Φ** are orthogonal to each other. Due to some entries of *f* are missing, the columns in **A** is shorter than the columns in **Φ**. and some columns of **A** are correlated.

Suppose there is another nonzero vector **x̃** ∈ ℝ*^d^* satisfying **Ax̃ = f***_a_* the support of **x̃** is *S***_x̃_** with |*S***_x̃_***|* = *k̃*. If the *i*th (*i* ∈ *S***_x_**) column in **A** is correlated with other columns, **f_a_** can also represented by weighted combinations of *k̃* column of **A** :

(9) $$f_{a}=\sum_{j\in S_{\tilde{\text{x}}}}{\tilde{x}}_{j}A_{j}$$

if $\sum_{j\in S_{\tilde{\text{x}}}}\left|{\tilde{x}}_{j}\right|\leq \sum_{j\in S_{\text{x}}}\left|x_{i}\right|$, the ℓ_1_-minimization algorithm will choose solution **x̃**, and thus leading to a wrong estimation.

Let **h** = **x** − **x̃**, then **Ah** = 0 meaning that **h** is a nonzero vector in the nullspace of **A**, and **h** has at most *k* + *k̃* nonzero entries. Because the sparsity-based interpolation method seeks the ℓ_1_ minimization solution under the constraint of available data consistency **Ax** = **Ax̃**, the error of interpolated signal solution **f̃** = **Φx̃** is:

(10) $${\left\|\tilde{\text{f}}-f\right\|}_{2}={\left\|\Phi \tilde{\text{x}}-\Phi \text{x}\right\|}_{2}={\left\|\text{B}\tilde{\text{x}}-\text{Bx}\right\|}_{2}={\left\|\text{Bh}\right\|}_{2}$$

When **Φ** is a basis, its rows are all orthogonal to each other, and the nullspace of **A** are spanned by the rows of **B**. So **h** is a linear combinations of rows of **B**, *i.e*., **h** = **B***^T^***α**, where **α** ∈ ℝ^*n*−*m*^. Then, Equation (9) can also be written as:

(11) $${\left\|\tilde{\text{f}}-f\right\|}_{2}={\left\|{\text{BB}}^{T}\text{α}\right\|}_{2}={\left\|\alpha \right\|}_{2}$$

Generally speaking, there may be multiple possible candidate solutions like **x̃** when the available samples are not enough. The best case is **x̃** = **x**, and **h** = 0 thus **α** = 0. The worst case is *S***_x_**∩*S***_x̃_** = ø, and **h** contains *k* + *k̃* ≤ *d* nonzero entries, and **α** has many nonzeros. When ‖**α**‖_2_ is smaller than expected error level, then we can say that we get a reasonable interpolation result.

Zhang *et al* [19] gave deterministic conditions that guarantee a successful exact recovery. It states the condition as strict *k* -balancedness of null space of **A**, where x^*^ is the sparsest solution to **f** = **Φx** with *k* nonzeros. Thus the following factors play important roles in recovery performance:

1. The sparser a vector **x** is, the more likely a null space {**v** ∈ ℝ^*n*^ : **Av** = 0} will be strict k-balanced.
2. Let **f***_a_* ∈ ℝ*^m^*, then the smaller *m* is, the less likely a null space will be strict *k* -balanced since the null space becomes larger.
3. The available data correspond to **A**, and the missing data correspond to **B.** In other words, the missing node locations decide the rows of **B**.
4. If **Φ** is a basis, the row vectors of **B** spans the null space.

However, the conditions for exact recovery are not verifiable in polynomial time. In this paper, we aim to reasonably interpolate the missing data, not necessarily to achieve exact recovery, so an important question is how to choose a good basis for data of sensor networks to get a more reasonable interpolation result?

From an application point of view, a WSN consists of spatially distributed autonomous sensors to cooperatively monitor physical or environmental conditions, such as temperature, sound, vibration, or pressure. Generally speaking, these physical phenomena are more often fields [13], so the network data are usually smooth. Due to the limited number of sensors, the data often have low resolution. Since the discrete cosine transform (DCT) can expresses a sequence of finitely many data points in terms of a sum of cosine functions oscillating at different frequencies, the DCT is an appropriate basis to sparsify WSN data which are smooth and in low-resolution. On contrary, natural images usually contain crisp boundaries or strong edges at localized regions. These image features can be sparsely represented by the localized basis such as wavelets.

According to Equation (8), let **A***_ij_* denote the *i*th entry of **A***_j_* (*j ∈ S***_x_**). Then, **A***_ij_* is the weight of *x_j_* for the linear combination $\sum_{j=1}^{d}A_{\mathrm{ij}}x_{j}$, where *i* corresponds to the *i*th available sample in **f***_a_*. *x_j_* has no way to be estimated if all its weights are zero. Let **Φ***_j_* be the *j*th column of dictionary **Φ**. If **Φ***_j_* has compact support and the missing block overlap with the compact support, then most of the entries in the **A***_j_* will be zeros.

**Figure 2.**
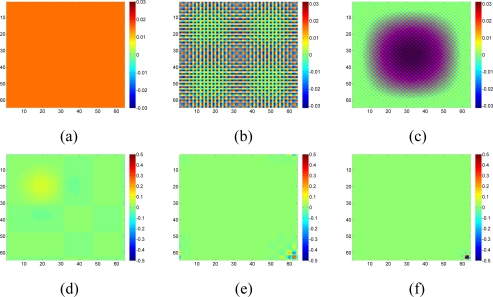
Basis functions of 2-D DCT and Wavelet, with size 64 × 64. **(a)**, **(b)** and **(c)** are DCT basis waveform according to its low, middle and high frequency component, respectively; **(d)**, **(e)** and **(f)** are Wavelet basis waveform according to its low, middle and high frequency component, respectively.

In this case, **A***_j_* cannot provide enough constraints that *x_i_* must satisfy. In another word, more nonzeros in **A_i_** can provide more information for *x_i_* because of more constraint equations. So, a dictionary with non compact support is preferred. If both DCT and wavelet transform can sparsify the data, DCT is a better choice since the wavelet basis functions are localized as Figure 2 shows, while DCT basis functions always have non-zero in a large range. If some parts of a DCT basis function are missing due to missing samples, the rest part of the function can still provide us information to recover the coefficients. Simulations in Figure 19 will demonstrate this issue.

## 3. Advantage

The IDWA interpolation assumes uniform correlation in the neighboring data. In many situations, this may not be true due to the fast and anisotropy changes in the neighborhood. A sparsity-based interpolation method does not require high correlation of the neighboring data. As long as the data are sparse in a chosen dictionary, it will work.

**Figure 3.**
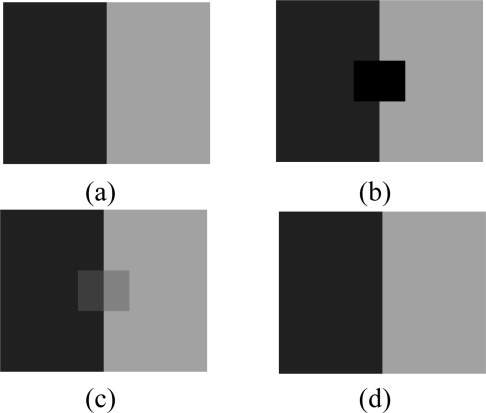
A toy example on boundary recovery. **(a)** Complete data, **(b)** Available data, **(c)** KNN interpolation, RMSE_KNN = 8.71 × 10^−2^, **(d)** Sparsity-based interpolation, RMSE_DCT = 4.31 × 10^−5^.

We created a toy example image like this: the left half of the image is a smooth image, while the right half is also smooth, but there is a sharp boundary. As shown in Figure 3, we can choose an artificial image for the left so that it is very sparse under DCT or wavelet domain. We could even simply linearly combine a few bases to form the left image. We construct a right half image similarly. Now suppose we only sample some pixels on the left half, and right half, we should be able to reconstruct the entire image nicely (e.g., the sparsity constraints select the basis functions that we used to generate the left image), but IDWA will blur the boundary. The sharp edge information is very hard for IDWA to capture because the weights of neighbor values depend on the distances between an interpolated node and its neighbors.

In the following, the K-nearest neighbor algorithm (KNN) [20,21] is chosen as an IDWA method for the 2-D case. The weight for each neighbor is computed by the inverse distance from the neighbor to the target missing samples. We use normalized root mean squared error (RMSE) to assess the accuracy of estimation which is defined as:

(12) $$\text{RMSE}\left(\text{f},\hat{\text{f}}\right)=\frac{\sqrt{\frac{\sum_{i=1}^{N}{\left(f_{i}-{\hat{f}}_{i}\right)}^{2}}{N}}}{f_{i_{\text{max}}}-f_{i_{\text{min}}}}$$

where *f_i_* and *f̂_i_* stand for *i*th (*i* = 1,2,⋯,*N*) entry of the original data vector **f** and the recovered data vector **f̂**, respectively. This normalization in RMSE allows for the comparison of estimation accuracy between different data sets.

## 4. A Fast Iterative Thresholding Algorithm to Solve the Sensor Data Recovery

Consider an optimization task that mixes ℓ_2_ and ℓ_1_ expressions in the form:

(13) $$F(\text{x})=\frac{1}{2}{\left\|f_{a}-\text{Ax}\right\|}_{2}^{2}+\lambda {\left\|\text{x}\right\|}_{1}$$

where *F*:ℝ*^N^* ↦ ℝ is a function of the vector **x**. This is a relaxed variant of the problem posed in Equation (6), and the parameter *λ* governs the tradeoff between the data consistency and the sparsity of **x**.

In recent years, a family of iterative thresholding algorithms has gradually been built to address the above optimization task in a computationally effective way [22–24]. Bredies and Lorenz [25] proves the convergence of iterative thresholding and they guarantees that the solution is the global minimizer for convex *F*(**x**). The core idea is to minimize the function *F*(**x**) iteratively [19], and Equation (7) can be simply solved by iterative thresholding:

(14) $${\text{x}}_{i+1}=S_{\lambda /c}\left(\frac{1}{c}A^{T}\left(\text{x}-{\text{Ax}}_{i}\right)+{\text{x}}_{i}\right)$$

where the parameter c will be chosen such that *c***I**−**A***^T^***A**>0 and *S*_λ/*c*_ (**τ**) is a soft thresholding operator to shrinkage each entry τ*_j_* of vector **τ** according to:

(15) $$S_{\lambda /c}\left(\tau_{j}\right)=\{\begin{array}{cc} 0 & ,\mathrm{if}\;\left|\tau_{j}\right|\leq \lambda /c\\ \tau_{j}-\frac{\left|\tau_{j}\right|}{\tau_{j}}.\frac{\lambda }{c} & ,\text{otherwise} \end{array}$$

**Figure 4.**
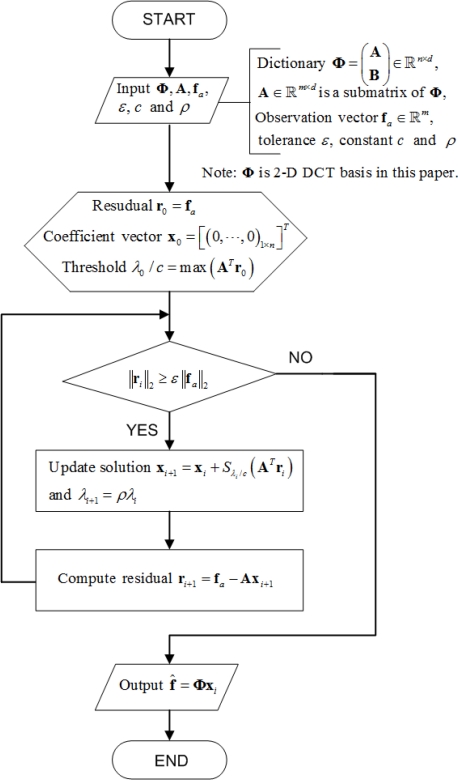
The flowchart for the sparsity-based interpolation algorithm with fast iterative thresholding.

However, the algorithm computes these solutions by updating the active set considering one coordinate at a time as a candidate to enter or leave the active set. Fadili *et al.* [26] demonstrated that using Equation (14) to solve Equation (7) can still be computationally demanding for large-scale problems, therefore we adapt here the same ideas and utilize a fast iterative-thresholding algorithms where the sequence *λ_i_* (*i* = 1,2,⋯) is allowed to be strictly decreasing. Figure 4 presents the flowchart of the soft iterative thresholding algorithm for sparsity-based interpolation.

The stop criterion *ɛ* depends on the fidelity of the received samples. The parameter *ρ* is adopted to decrease the threshold *λ_i_*/*c* in each iteration. The smaller *ρ* is, the faster **x** converges. The two parameters *η* and *ρ* in the algorithm are constants, and we set them to be the same in all the experiments. From empirical analysis, *ɛ* = 10^−9^ and *ρ* = 0.95 give good results for our experiments.

## 5. Simulation and Analysis

In this section, we provide some numerical simulations for 1-D and 2-D missing data recovery. In order to validate the proposed approach, we generate 1-D and 2-D synthetic data which can be sparsely represented as DCT coefficients, and compare the estimation accuracy of the proposed approach with IDWA interpolation on these synthetic data sets. We also use real sensor data sets [2] to validate our method. All the simulation results with the sparsity-based data interpolation are accomplished only with DCT as the sparsifying transform, except the simulation in Figure 19 where the failure of wavelets is shown for real data. We do not compare our method with the Kriging method since no historical variogram is available in our experimental data.

All the simulations are repeated 100 times, and the locations of missing samples are changed for each repeated simulation. The average and standard deviation of RMSE are computed. The main parameters in the simulations are *N*, the number of samples in the original data; *M*, the number of missing samples; and *S*, the number of nonzero sparse coefficients. We also define the missing rate as $m=\frac{M}{N}$ and sparsity $s=\frac{S}{N}$. The relationships among estimation accuracy different missing rates and missing square block sizes are discussed in the next sections.

### 5.1. Experiment with Synthetic 2-D Data

In this subsection, the KNN and the sparsity-based interpolation methods are compared on the synthetic data. Sensor data are smooth if they have a strong spatial correlation. By applying the 2-D DCT transform on the spatially deployed sensor data, the major energy of these data will concentrate on low frequency domain. When the data change rapidly in local regions, it has strong high frequency components in the DCT domain. So, it is meaningful to discuss the performance of missing data recovery when the sparsity of sensor data is represented in the high frequency, low frequency, and mixed high and low frequency DCT coefficients, respectively. In addition, what the recovered data look like for different missing patterns and missing rates is also very useful. The effect of the missing-block-size on the RMSE is also discussed.

**Figure 5.**
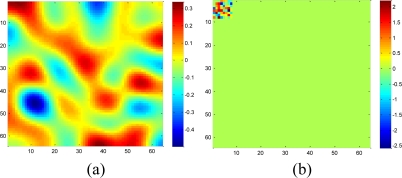
2-D synthetic low frequency data with size 64 × 64 is generated from 64 nonzero coefficients in low frequency domain of DCT. **(a)** 2-D synthetic data. The color bar denotes the sample value of each spatial node. **(b)** 64 nonzero coefficients in DCT dictionary. The size of the DCT dictionary is 64 × 64. The color bar denotes the coefficient value of each atom in DCT dictionary.

**Figure 6.**
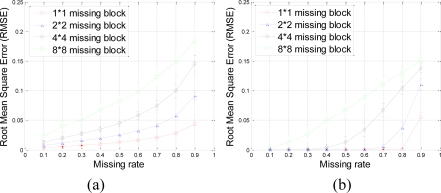
Effect of missing rate and missing block size on estimation quality with spatially smooth sensor data. **(a)** and **(b)** shows the RMSE curve of KNN and the proposed approach, respectively. Error bar stands for the standard deviation with aspect to the repeated 100 times of simulations for the same size of missing blocks and same missing rate. This can help eliminating and understanding the influence of randomness of each simulation.

A set of 64 × 64 2-D synthetic data, shown in Figure 5(a), is generated from 64 nonzeros in low frequency DCT domain as shown in Figure 5(b). It is clear that the low frequency DCT coefficients can provide a smooth representation of spatially 2-D sensor data. Figure 6 shows the recovery performance of KNN and the proposed approach for spatially smooth data. KNN results in a large RMSE which means KNN fails to recover the missing samples. When a block of samples are missing, KNN has to choose the nodes beyond the block as its neighbors whose values may differ significantly from the interpolated node, indicating that KNN is sensitive to the size of missing patterns.

Our method produces very low RMSE if the missing-block-size is smaller than 8 × 8 and the missing rate is smaller than 0.5. This missing rate is promising since we can recover the missing samples when half of sensor data are missing. As the missing rate increases, the RMSE of the proposed method remains nearly the same within certain intervals until the missing rate reaches a turning point.

As shown in Figure 6(b), for example, if the acceptable value of RMSE is at 10^−4^, the turning point of missing rates are 0.3, 0.6, and 0.8 for 4 × 4, 2 × 2, and 1 × 1 missing-block-sizes, respectively. It is a very appealing characteristic that in these stable ranges, the estimation quality is still good and nearly independent of the missing rate. This can be explained by Equation (7), which states that when the number of samples is large enough, the missing samples can be well recovered with overwhelming probability. The stable range shortens as the missing-block-size increases because the increase of block-size introduces less randomness to the sensing matrix **Φ**. When a large block of sensor samples are missing, e.g., 8 × 8, the proposed method cannot guarantee a low RMSE. However, 8 × 8 missing-block is a very extreme case for the 64 × 64 sensor network. Even in this situation, our method performs better than KNN in term of the RMSE.

**Figure 7.**
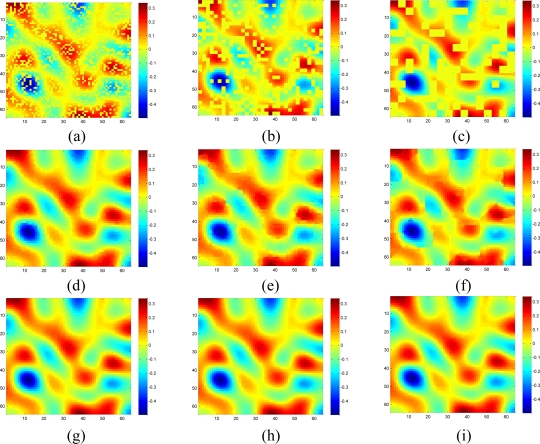
Effect of missing block size on the estimation quality of Figure 5(a) for missing rate = 0.3. **(a–c)** show sensor data with 1 × 1, 2 × 2 and 4 × 4 missing block size, respectively; **(d–f)** Recovered data by KNN under (a–c) respectively; **(g–i)** Recovered data by the proposed approach under (a–c), respectively.

Figure 7 shows the recovered data by KNN and the proposed approach under different missing–block-sizes. The missing rate is fixed at 0.3. We can see that the estimation quality is much better by our sparsity-based recovery method than KNN. For KNN, due to the missing blocks of samples, the recovered data suffer from blocking effects, *i.e.*, the edge is not smooth for the missing-block. This effect becomes worse when the missing-block-size increases. Conversely, the proposed approach recovers the missing data almost equally well under different missing-block-sizes.

**Figure 8.**
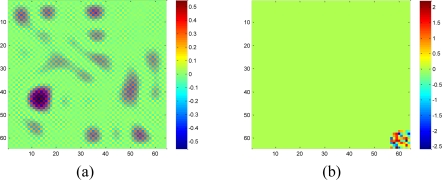
2-D synthetic high frequency data with size 64 × 64 is generated from 64 nonzeros coefficients in high frequency domain of DCT. **(a)** 2-D synthetic data. The color bar denotes the sample value of each spatial node. **(b)** 64 nonzero coefficients in DCT dictionary. The size of the DCT dictionary is 64 × 64. The color bar denotes the coefficient value of each atom in DCT dictionary.

**Figure 9.**
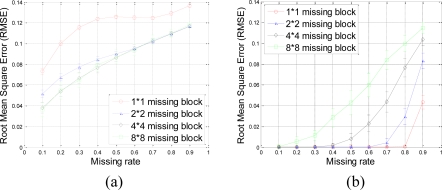
Effect of missing rate and missing block size on estimation quality with oscillating sensor data. **(a)** and **(b)** shows the RMSE curve of KNN and the proposed approach, respectively. Error bar stands for the standard deviation with aspect to the repeated 100 times of simulations for the same size of missing blocks and same missing rate. This can help eliminating and understanding the influence of randomness of each simulation.

Now, we discuss the performance of recovery for fast changes in the sensor data network. The 64 × 64 2-D synthetic data, shown in Figure 8(a), is generated from 64 nonzero high frequency DCT coefficients as shown in Figure 8(b). In this case, fast oscillations are presented. Figure 9 shows the recovery performance of KNN and the proposed approach for oscillating data. In Figure 9(a), KNN performs poorly in term of the RMSE. Because KNN has the tendency to smoothen the data, it is not suitable for high frequency data. Under different block sizes, the RMSE curves of KNN for high frequency data are all much higher than that for low frequency data. On the contrary, our method approaches very low RMSE if the block-size is smaller than 8 × 8 and the missing rate is smaller than 0.5. This result is very similar to the recovery of smooth data in Figure 6(b). So, if the sparsity is satisfied and the block-size is not too large, the sparsity-based recovery is robust to smooth or oscillating sensor data. According to the compressive sensing theory, sparsity-based recovery mainly depends on the global sparsity of data but not too much on whether this data is sparse in low or high frequencies.

Meanwhile, the proposed approach can get good estimation results as long as the missing rate is lower than a certain value, e.g., 0.3, 0.6, and 0.8 for 4 × 4, 2 × 2, and 1 × 1 missing-block-size, respectively. Thus, the proposed approach is robust with different sizes of missing blocks. For example, with 4 × 4 missing-blocks, the RMSE is still low if the missing rate is smaller than 0.3. It means one quarter of sensors samples can be missed although the block-size is a little large for a 64 × 64 sensor network.

However, an interesting phenomenon in Figure 9(a) is that larger missing blocks lead to lower RMSEs of KNN. Due to the periodic oscillation of the synthetic high frequency data, when missing block is larger than one period of cosine wave, at least one missing sample is recovered correctly. While missing block is small, it is hard or even impossible to recover any missing samples, e.g., each of them cannot be represented via the linear combination of its nearest neighbors.

**Figure 10.**
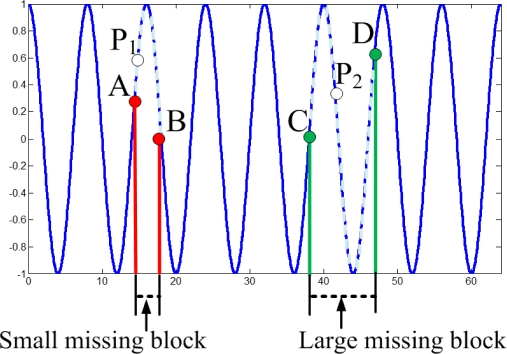
Missing sample estimation of a high frequency component with KNN. The solid line represents available samples, where the dash line denotes missing samples. Points **A**, **B** are nearest neighbors of a small missing block, and **C** and **D** are nearest neighbors of a large missing block. Points P1, P2 are missing points to be recovered.

An intuitive explanation is shown in Figure 10 for the 1-D high-frequency component which is generated from high-frequency DCT coefficients. For the small missing block, suppose the value of point P_1_ is missed, A and B are the nearest neighbors of P_1_, then P_1_ is hard to be recovered via linear combination of A and B. However, for the large missing block, suppose the value of point P_2_ is missed, C and D are the nearest neighbors of P_2_, then it is possible to recover P_2_ via the linear combination of C and D. This implies large block size may help KNN to recover the missing samples for the high-frequency data. This can explain why larger missing blocks lead to lower RMSEs.

**Figure 11.**
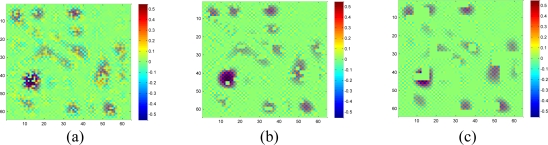

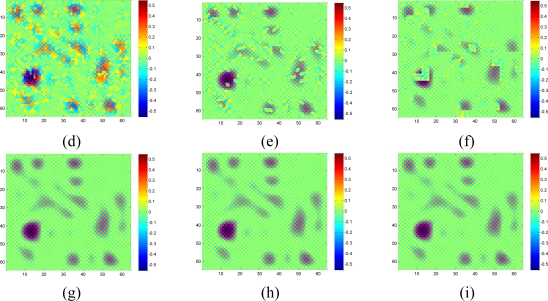
Effect of the missing block size on the estimation quality of Figure 8(a) for missing rate at 0.3. **(a), (b) and (c)** show sensor data with 1 × 1, 2 × 2 and 4 × 4 missing block size, respectively; **(d), (e) and (f)** are the recovered data by KNN corresponding to (a), (b) and (c), respectively; **(g)**, **(h) and (i)** are the recovered data by the proposed approach corresponding to (a), (b) and (c), respectively.

Figure 11 shows the recovered high frequency data by KNN and the proposed approach under different missing-block-sizes. The missing rate is fixed at 0.3. Obviously, the new method outperforms KNN since KNN fails to recover the data while our method successfully recovers the missing data for different missing-block-sizes.

**Figure 12.**
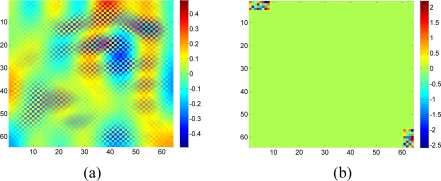
2-D mixed data with size 64 × 64 is generated from 32 nonzeros of low frequency DCT coefficients and 32 nonzeros of high frequency DCT coefficients. **(a)** Original data. **(b)** DCT coefficients. The size of the DCT dictionary is 64 × 64. The color bar denotes the coefficient value of each atom in DCT dictionary.

Meanwhile, the real WSN data always contain both low frequency components and high frequency components simultaneously. So a 2-D synthetic data with size 64 × 64 is generated from 32 nonzero coefficients in the low frequency DCT domain and 32 nonzero coefficients in the high frequency domain, which is shown in Figure 12.

**Figure 13.**
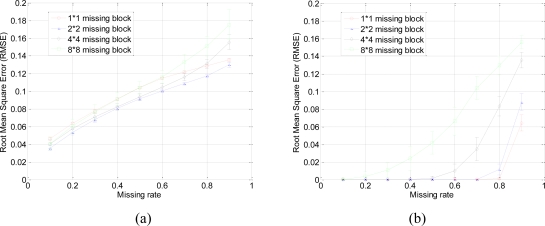
Effect of missing rate and missing block size on estimation quality with mixed sensor data. **(a)** and **(b)** shows the RMSE curve of KNN and the proposed approach, respectively. Error bar stands for the standard deviation with aspect to the repeated 100 times of simulations for the same size of missing blocks and same missing rate. This can help eliminating and understanding the influence of randomness of each simulation.

**Figure 14.**
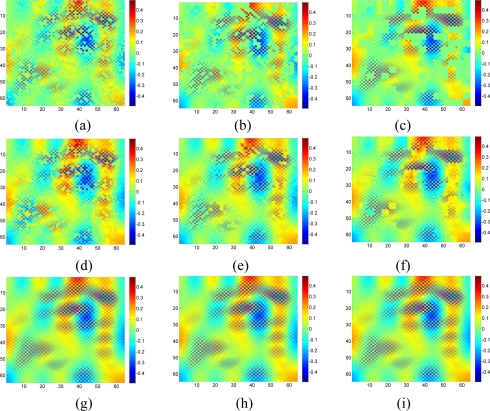
Effect of the missing-block-size on the estimation quality of Figure 11(a) for missing rate=0.3. **(a), (b) and (c)** show sensor data with 1 × 1, 2 × 2 and 4 × 4 missing block size, respectively; **(d)**, **(e) and (f)** are the recovered data by KNN corresponding to (a), (b) and (c), respectively; **(g)**, **(h) and (i)** are the recovered data by the proposed approach corresponding to (a), (b) and (c), respectively.

The recovery RMSE curves of KNN and the proposed approach for the mixed data are shown in Figure 13. Meanwhile, fixing the missing rate at 0.3, Figure 14 compares the visual recovered data by these two methods under different missing–block-sizes. The result on mixed data is in accordance with the simulations on low and high frequency components separately. The only difference is the RMSE curves of KNN under different size of missing blocks become closer to each other. The reason is that when block size becomes larger, KNN’s RMSE increases for low frequency components, but decreases for high frequency components. Now since mixed signal contain both low and high frequency components, the two opposite effects cancel out each other.

From the above simulations on recovering the missing samples of smooth, oscillating and mixed sensor data, it is clear that the proposed method can successfully recover the missing samples if the sensor data can be sparsely represented in a transform domain and the number of available samples is enough. Specifically, the proposed approach is much more robust to both the block-missing-size and missing rate than the conventional weighted averaging method such as KNN. And it does not rely on locations of nonzero DCT coefficients since the *l*_1_ norm is separable.

### 5.2. Experiment with Real 2-D Data

**Figure 15.**
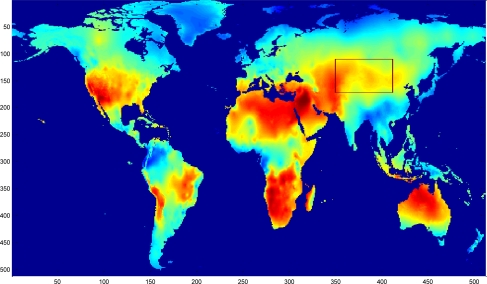
A snapshot of mean monthly surface sunshine duration in June over global land areas, excluding Antarctica.

To validate the performance of the sparsity-based missing data recovery in a sensor network, a mean monthly surface climate over global land areas, excluding Antarctica [2] is employed as the data set for simulation. The climatology data includes eight climate elements—precipitation, wet-day frequency, temperature, diurnal temperature range, relative humidity, sunshine duration, ground frost frequency and wind speed—and was interpolated from a data set covering the period from 1961 to 1990. The data are available through the International Water Management Institute World Water and Climate Atlas (http://www.iwmi.org) and the Climatic Research Unit (http://www.cru.uea.ac.uk). This data set consists of the monthly averaging surface sunshine duration in June over global land areas from 1961 to 1990. The final measurement points in the data set formed a regular grid of 10’ latitude/longitude over the region under study. We select a subset of 64 × 64 data that has no missing values, shown in Figure 15, as the original data without missing samples.

Since the data are the average values from 1961 to 1990, it is very smooth and should be highly compressible in the DCT domain. When applying the real data set to simulate the sparsity-based signal processing, Luo *et al.* [27] suggest preserving the *S* largest coefficients in a transform domain. Let **α** = [*α*_1_ *α*_2_ ⋯ *α_N_*]*^T^* be a vector to represent the DCT coefficient of the real data, we define **α**_*s*_ as the vector of coefficients (*α_i_*) with all but the largest *S* set to zero. By calculating the normalized energy loss that is smaller than 10^−5^:

(16) $$\frac{{\left\|\alpha -\alpha_{S}\right\|}_{2}^{2}}{{\left\|\alpha \right\|}_{2}^{2}}<10^{-5}$$

**Figure 16.**
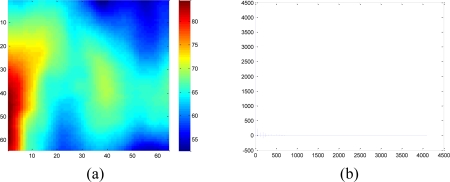
A 64 × 64 2-D smoothed real dataset and its DCT coefficients, which keeps more than 99.99% energy of raw data. **(a)** 2-D real dataset. **(b)** DCT coefficient vector contains 240 nonzeros. The size of the DCT dictionary is 64 × 64. The color bar denotes the coefficient value of each atom in DCT dictionary.

S = 240 is achieved for the selected subset dataset. Equation (16) shows that keeping largest 240 coefficients leads to little loss of energy and preserves most information of **α**. Figure 16 shows the smoothed real data by keeping 240 largest coefficients and set remaining ones zero. This makes sense since most real signals can be represented with a few coefficients in a transform domain without losing much information [28,29].

**Figure 17.**
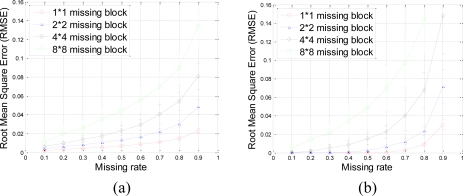
Effect of missing rate and missing block size on estimation quality with real sensor data. **(a)** and **(b)** show the RMSE curve of KNN and the proposed approach, respectively. Error bar stands for the standard deviation with aspect to the repeated 100 times of simulations for the same size of missing blocks and same missing rate. This can help eliminating and understanding the influence of randomness of each simulation.

The RMSE performance of this data set is evaluated in terms of missing block size and missing rate in Figure 17.

Our proposed method results in very small RMSE when the missing rate is smaller than 0.5 for 1 × 1 and 2 × 2 missing-blocks. The improvement over KNN holds for 1 × 1, 2 × 2 and 4 × 4 blocks until too many sensor samples are missing, *i.e.*, when the missing rate is larger than 0.8. Both our proposed method and KNN have very large RMSE for 8 × 8 missing-blocks because the missing-block-size is too large for the 64 × 64 sensor network.

**Figure 18.**
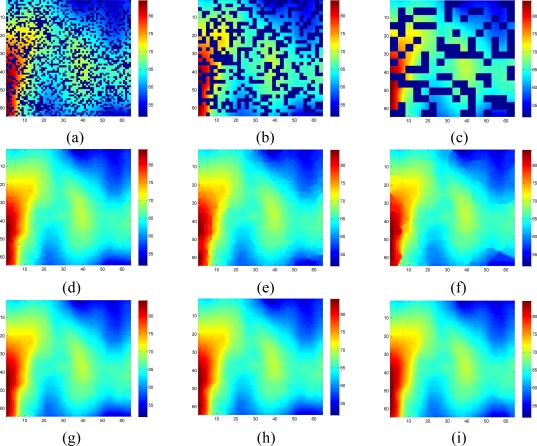
Effect of the missing block size on the estimation quality of Figure 16(a) for missing rate at 0.4. **(a)**, **(b)** and **(c)** show sensor data with 1 × 1, 2 × 2 and 4 × 4 missing block size, respectively; **(d)**, **(e)** and **(f)** are the recovered data by KNN corresponding to (a), (b) and (c), respectively; **(g)**, **(h)** and **(i)** are the recovered data by the proposed approach corresponding to (a), (b) and (c), respectively.

Figure 18 shows the recovered sensor network data with a missing rate 0.4. Compared with the non-missing data in Figure 16(a), KNN failed to recover some features and introduce the blocking artifacts when the missing block becomes large. As shown in Figure 18(d–f), this disadvantage of KNN becomes serious when the missing-block-size increases. In contrast, our method shows the ability to recover the data without significant information loss. In addition, the change of missing-block-size nearly does not affect the recovered data. Thus, the proposed method is robust to missing block sizes.

As demonstrated from Figures 5 to 14, the proposed method can give much better performance in simulations. However, for the real dataset captured from a snapshot of mean monthly surface sunshine, these advantages are not so evident, as shown in Figures 17 and 18. According to the simulation results on both synthetic data and real data, the estimation quality of the proposed method are lowered with the increase of the value *M*/*S*, where *M* denotes missing rate, and *S* denotes the number of nonzero coefficients. Fixing *M* and total number of data *N = 64 × 64*, a smaller *S* will produce better performance, or lower RMSE. The three synthetic data sets in Figures 5, 8, 12 all have *N = 64 × 64* and *S = 64*, and the real data set in Figure 16(a) has *N = 64 × 64* and *S = 240*, which means it has more nonzero coefficients. That is why the advantages are not so evident on the real data as on the synthetic data. However, one can still observe the proposed method can overcome the obvious blocky artifact of KNN in Figure 18. What is more, if sensor data contain fruitful high-frequency components, e.g., rapid changes in localized regions, the advantage of the proposed method will become more obvious, as demonstrated in the results of synthetic high frequency data.

### 5.3. Comparisons of DCT and Wavelets for the Sparsity-Based Interpolation

**Figure 19.**
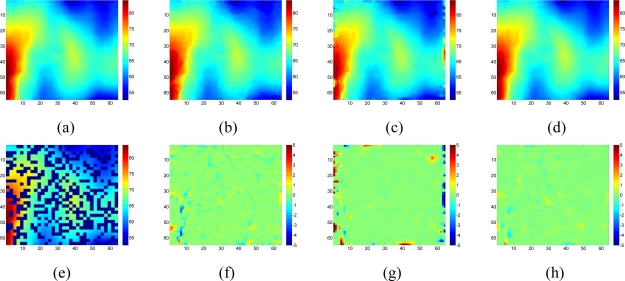
Comparisons on the wavelet-based, DCT-based sparsity-interpolation and KNN interpolation for the data represented by sparse wavelet coefficients. **(a)** A 64 × 64 2-D smoothed real dataset in wavelet domain, which keeps more than 99.99% energy of raw data, **(b)** recovered data by KNN, **(c)** recovered data by proposed method with wavelet, **(d)** recovered data by proposed method with DCT, **(e)** available data when missing rate is 0.4 and block size is 2 × 2, **(f)**, **(g)** and **(h)** are the recovered error of (b), (c) and (d), respectively, and the RMSE of three methods are 0.31, 0.99 and 0.18, respectively.

Now suppose we choose a 2-D wavelet as the sparsifying transform for the real data, and by calculating the normalized energy loss as Equation (16), the largest *S = 408* wavelet coefficients are kept and the rest wavelet coefficient are set to be zeros. Figure 19 compares the estimation quality of the real data by KNN, the proposed method with wavelet or DCT dictionaries.

Although the data is composed of sparse wavelet coefficients, obvious recovery errors are observed for the wavelet-based recovery samples as shown in Figures 19(c,g). As we explained in the Section 2, when the relatively large missing blocks overlaps with the compact support of wavelet basis, most of the weights in the underdetermined equations in Equation (8) will be 0. Thus, not enough information is taken use of by using the wavelet basis to recover the data. On the contrary, DCT produces the lowest RMSE in the three methods. Since DCT do not have the localized support, less entries in the underdetermined equations in Equation (8) will be nonzero, this can provide more information than wavelet to help recover the missing samples. Therefore, a non compact support basis is preferable for the spatial interpolation.

## 6. Conclusions and Future Work

In this paper, we have proposed a sparsity-based method to recover the missing data in wireless sensor networks. Instead of investigating the correlation in local neighboring sensors, the proposed approach exploits the sparsity of network data by solving the *l*_1_ norm minimization problem. Both synthetic and real data simulation results demonstrate that the proposed approach can successfully recover the missing data and that there exists a flexible range of missing rates where the proposed method is robust to missing block size, as long as the network data have the sparsity property.

Although the sparsity-based interpolation shows advantages over KNN, for some other applications and under different assumptions, it could be wed with KNN as well as other interpolation methods to make full use of their respective advantages. For example, one limitation of sparsity-based interpolation is that the number of available samples should be enough to estimate the DCT coefficients. Based on the observation that KNN can recover the missing samples reasonably when the data only contains low-frequency components and size of missing blocks is not too large as shown in Simulation part, KNN could be utilized to estimate the low-frequency components and sparsity-based interpolation is employed to estimate the high-frequency components. This potentially requires less available samples for the sparsity-based method since the unknowns for *l*_1_ minimization are reduced. For the future work, an extension of the proposed method for the irregular grid by dividing the whole network field into cells will be further investigated. Also we will extend it to 3-D case where the third dimension is time.
